# Facile synthesis of FeS–Fe_3_O_4_ nanocomposites: highly stable & enhanced electrochemical performance in asymmetric supercapacitor applications

**DOI:** 10.1039/d5na01165e

**Published:** 2026-02-17

**Authors:** Junaid Riaz, Zahra Bayhan, Ghulam Murtaza, Muhammad Arif, Amina Bibi

**Affiliations:** a Yunnan Key Laboratory of Optoelectronic Information Technology, School of Physics and Electronic Information, Yunnan Normal University Kunming 650500 China junaidriaz1990@gmail.com; b Department of Physics, College of Sciences, Princess Nourah bint Abdulrahman University P.O. Box 84428 Riyadh 11671 Saudi Arabia; c School of Ecology and Environmental Science, Yunnan University, Biocontrol Engineering Research Center of Crop Diseases & Pests Kunming 650500 Yunnan Province China; d Department of Physics, Hazara University Mansehra 21300 Pakistan aminaamni11@gmail.com

## Abstract

Over the last decade, researchers have required electrode materials that have higher energy density and are highly stable to make the next generation of supercapacitors. This study developed and analyzed a FeS–Fe_3_O_4_ nanocomposite as a potential positive electrode material for asymmetric supercapacitors. Structural and morphological analyses were performed using XRD and FESEM, with EDX and EDS mapping. The FeS–Fe_3_O_4_ composite worked better than both pure FeS and pure Fe_3_O_4_ when tested with a 3 M KOH electrolyte. At 1 A g^−1^, it has a specific capacitance of 464.6 F g^−1^, and at 4 A g^−1^, it has a specific capacitance of 186.9 F g^−1^. The better performance is because FeS conducts electricity better, and Fe_3_O_4_ acts like a pseudocapacitor. These two factors work together to speed up the movement of ions and the redox processes. The supercapacitor fabricated in this work exhibits a unique configuration that has never been reported before. The negative electrode was made of activated carbon, and the positive electrode was made of FeS–Fe_3_O_4_. It can work within a voltage window of 1.4 V. The device had a power density of 699.6 W kg^−1^ and an energy density of 46.10 Wh kg^−1^. It also delivered a high power density of 3998.4 W kg^−1^, with a low energy density of 30.17 Wh kg^−1^. After 12 000 charge–discharge cycles, the device retained 98.6% of its capacitance and about 99.8% coulombic efficiency, which indicates good cycling stability. The results show that the FeS–Fe_3_O_4_ nanocomposite is a very good electrode material for energy storage systems that need to be stable and work well.

## Abstract 2

Over the last decade, researchers have required electrode materials that have higher energy density and are highly stable to make the next generation of supercapacitors.

## Graphical abstract

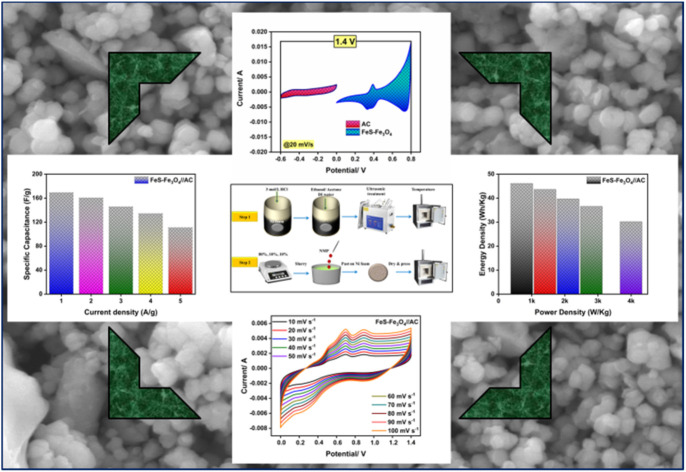

## 1. Introduction

In the last few decades, industrialization and fast economic growth have caused excessive use of fossil fuels, which has played a big role in global warming.^1–4^ It is important to create energy technologies that are both long-lasting and affordable as society moves into the next phase in order to meet the needs of modern living and industry. The rising demands of manufacturing, mechanization and the global economy are driving an increase in energy demand.^5–8^ This increased demand for renewable energy sources is vital for preserving global ecosystems. Without the use of renewable energy, it will be impossible to halt the depletion of the ozone layer.^9–12^ Developing a clean, safe, and efficient system for energy conversion and storage is crucial. Supercapacitors show great potential in addressing the energy storage requirements of electronic devices, including hybrid and electric vehicles (EVs and HEVs). Their fast charging ability, high power density, and long cycle life make them ideal for applications that require rapid energy release. Their versatility in supplementing or replacing batteries in certain situations further enhances their value in the development of advanced energy storage technologies.^13–19^ Supercapacitors are superior to conventional capacitors because they can store more energy, work in a wider range of temperatures, and have a higher specific capacitance. The capacitance in supercapacitors increases due to the interaction between the electrolyte and the electrode material on nickel foam, or it can arise from the faradaic electron charge generated during redox reactions or intercalation processes. Faradaic reactions make charge transfer easier.^20^ A lot of researchers are interested in hybrid supercapacitors (HSCs) since they perform very well when electric double-layer capacitors (EDLCs) and pseudo-capacitors (PCs) are coupled. The electrochemical characteristics and capacitance are enhanced when faradaic redox processes are combined with double layers.^21–23^ The type of material used for the electrode has a huge effect on how well HSCs perform, similar to batteries. This is because the energy density, power density, and cycle stability are affected.^24–26^ Electric double-layer capacitors (EDLCs) store charge through ion adsorption at the electrode–electrolyte interface, typically using carbon-based materials, offering high power density and long-term cycling stability. In contrast, pseudocapacitors (PCs) store energy *via* fast faradaic redox reactions in electroactive materials such as metal oxides or conducting polymers, providing higher capacitance. Hybrid supercapacitors combine EDLC and pseudocapacitive electrodes to achieve improved energy density and rate capability.^27–29^ Because of these qualities, metal sulfides and other metal compounds, such as metal oxides, are thought to be some of the best materials for electrodes in the next generation of supercapacitors.^30^

Transition metal oxides (TMOs), such as ZnO, Fe_2_O_3_, NiO, CoAl_2_O_4_ and Fe_3_O_4_, have been widely researched as effective electrode materials for supercapacitors, offering higher capacitance than conventional electric double-layer capacitors (EDLCs).^31–35^ These TMOs utilize redox reactions to provide superior specific energy compared to traditional carbon-based materials, and they also show greater durability than conducting polymers.^36–38^ Iron oxides are well known for their excellent electrochemical redox characteristics, high capacity, environmental friendliness, abundance, non-toxicity, and low cost.^39^ Iron oxides can store charge across a wide voltage range because Fe^3+^ and Fe^2+^ can undergo reversible oxidation and reduction. However, iron oxides, specifically Fe_3_O_4_, have relatively low conductivity and stability, which limits their electrochemical performance and cycling stability.^40^

Magnetite (Fe_3_O_4_) is one of the transition metal oxides that has been found to have excellent conductivity and high electrochemical activity. This substance is appealing since it is abundant in nature, safe for people and the environment, and has little effect on the environment.^41^ Wang *et al.* examined the electrochemical performance of Fe_3_O_4_ nanoparticles exhibiting a high surface area (165.05 m^2^ g^−1^) produced *via* a hydrothermal process.^42^ Reyes *et al.* created Fe_3_O_4_ nanoparticles that have a specific capacitance of 36 F g^−1^.^43^ The specific capacitance of Fe_3_O_4_ is still lower than that of other transition metal oxides, though. Iron oxides are particularly important among transition metal oxides because of their environmental sustainability and abundant natural reservoirs, along with intrinsic pseudo-capacity properties, making them an outstanding choice for faradaic pseudo-capacitors. Insufficient electrical conductivity and particle agglomeration during charge/discharge cycles, on the other hand, limit Fe_3_O_4_, causing a sharp drop in capacity at elevated current densities and following extended cycling.^44–48^ Chang *et al.*^49^ prepared Fe_3_O_4_/graphene nanocomposites through a solvothermal process and obtained a specific capacitance of 300 F g^−1^ at 0.4 A g^−1^. Regardless of such improvements, there are still limitations to the practical application of Fe_3_O_4_-based materials including short cycle life, rate capability, and low electrical conductivity that impede their large-scale utilization.^50^ Iron sulfide (FeS) meets all the important requirements for supercapacitor electrode materials, such as being cheap, plentiful, and non-toxic. In the last few decades, many people have worked on making FeS nanosheets so that they can take advantage of these benefits. It is very important to make new electrode materials like FeS since they are in great demand and cheap, and have a large surface area. These factors are important for making supercapacitor technology more commercially viable.^51^

This study focuses on the development of hybrid electrode materials that effectively combine high electrical conductivity with significant redox activity, suggesting a promising yet complex strategy to improve supercapacitor performance. TMS and TMO have attracted significant interest due to their synergistic electrochemical capabilities; yet, their specific restrictions often hinder practical applications. To address these challenges, this study demonstrates the systematic fabrication of a FeS–Fe_3_O_4_ nanocomposite, in which the synergistic interaction between conductive FeS and pseudocapacitive Fe_3_O_4_ markedly enhances charge storage performance. The FeS–Fe_3_O_4_ nanocomposite has a high specific capacitance of 464.6 F g^−1^ at 1 A g^−1^ and preserves its outstanding rate capability because its porous structure is well-integrated. When used to create an asymmetric supercapacitor using activated carbon as the negative electrode, the device operates over a broad voltage range of 1.4 V. It has a high energy density of 46.10 Wh kg^−1^ and can retain 98.6% of its capacitance after 12 000 charge–discharge cycles, which indicates good cycling stability. These results demonstrate that FeS–Fe_3_O_4_ nanocomposites could be highly beneficial as electrode materials and represent an excellent approach to develop energy storage devices that perform well and have long-term stability.

## 2. Experimental section

### 2.1 Materials for experiments

All materials were purchased from Sigma-Aldrich (China) and used in the experiments without further treatment or purification: ferrous sulfate heptahydrate (FeSO_4_·7H_2_O), iron(iii) chloride hexahydrate (FeCl_3_·6H_2_O), thioacetamide (C_2_H_5_NS), ammonia solution (NH_3_·H_2_O), sodium hydroxide (NaOH), ethanol (C_2_H_5_OH), hydrochloric acid (HCl), *N*-methyl-2-pyrrolidone (NMP), polyvinylidene (PVDF), acetone and DI water.

### 2.2 Hydrothermal synthesis of Fe_3_O_4_

To prepare Fe_3_O_4_ by using the hydrothermal method, 0.1 mmol solution of ferrous sulfate (FeSO_4_·7H_2_O) was added to 40 ml DI water. The mixture was placed on a hotplate stirrer for 3 h at room temperature. The pH of the solution was maintained at 10 by adding ammonia solution dropwise into the solution during heating at 80 °C for 40 min, due to which the color of the solution changed from bright green to black. Then the solution was transferred into an autoclave and put into a furnace at 80 °C for 4 h. After cooling naturally to room temperature, the particles were washed by centrifugation at 4000 rpm until their pH reached 7. After washing the sample many times, the sample was put into an oven for drying at 60 °C overnight to remove the water residue.

### 2.3 Hydrothermal synthesis of FeS

For the preparation of FeS, 6.8 g of C_2_H_5_NS and 5.8 g of FeCl_3_·6H_2_O were dissolved under magnetic stirring in 15 ml of DI water and 5 ml of ethanol. After 30 minutes of magnetic stirring at room temperature, the beaker was subjected to ultrasonic treatment for the next 40 minutes for homogenous mixing. Then the prepared solution was transferred to a 30 ml stainless autoclave and heat-treated for the next 2 h at 150 °C. The thermal treatment in the autoclave facilitates good crystallinity and the desired morphology. After cooling to room temperature in an open atmosphere, the sample was washed by centrifuging many times and then put into an oven at 70 °C for the next 4 h.

### 2.4 Preparation of FeS–Fe_3_O_4_ composites

For the preparation of FeS–Fe_3_O_4_ composites *via* wet chemical method, we take the weight ratio of our prepared samples, FeS, and Fe_3_O_4_, and dissolved them in 20 ml of DI water by adding sodium hydroxide using a magnetic stirrer, at 60 °C for 2 h. After that, we provide the ultrasonic treatment for 40 minutes to disperse our materials. And then put into the oven for drying at 80 °C overnight, and get the FeS–Fe_3_O_4_ composite with enhanced electrochemical performance, then pure FeS, and Fe_3_O_4_ materials.

### 2.5 Electrode preparation process

**Fig. 1:**
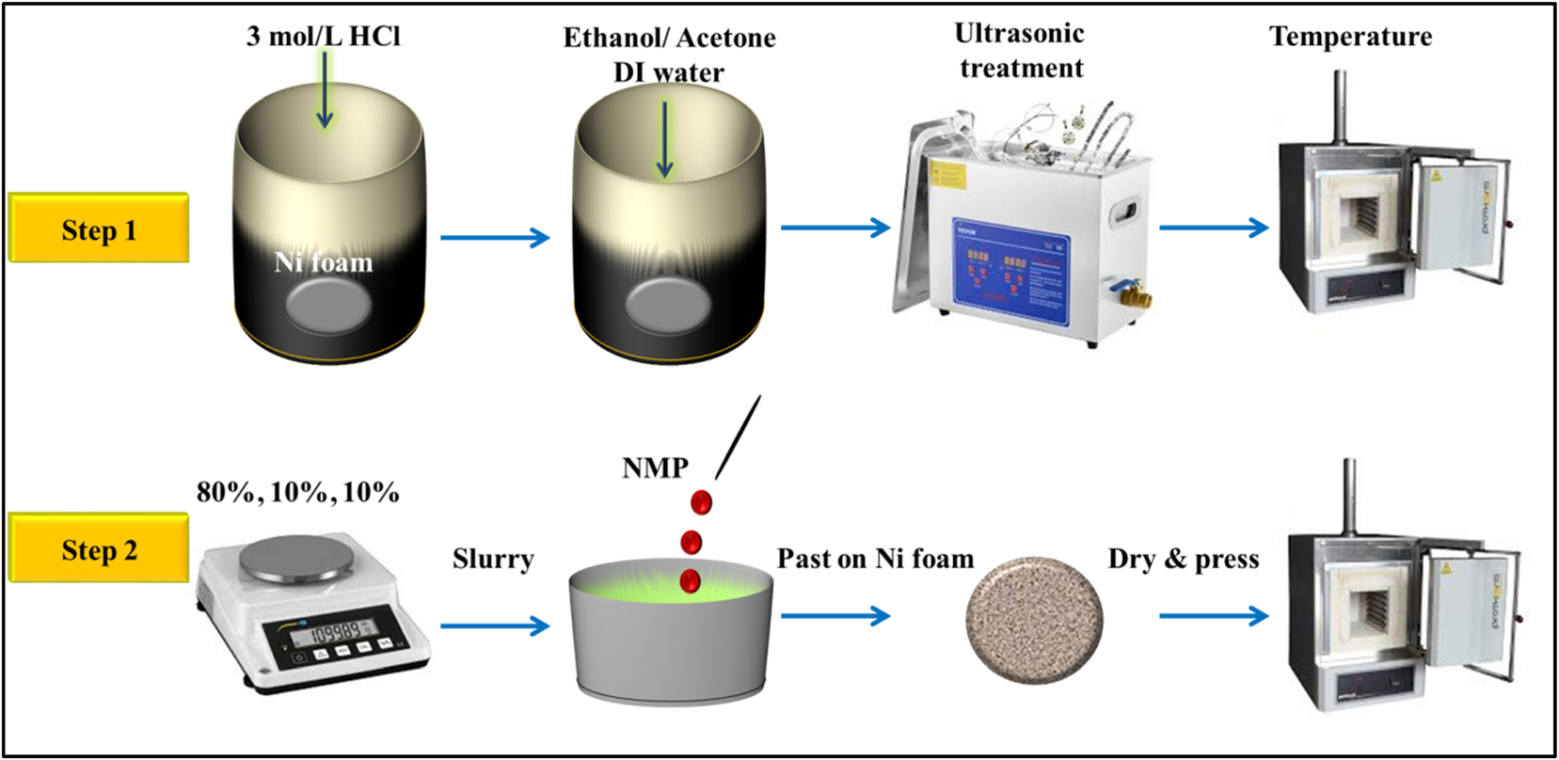
Preparation method for FeS, Fe_3_O_4_, and Fes–Fe_3_O_4_ composite electrodes.

For the preparation of Fe_3_O_4_, FeS, and FeS–Fe_3_O_4_ composite electrodes, first, we cut the Ni-foam into a 1 × 1 cm circular shape, washed it with acetone, 3 M HCl, 100% ethanol, and DI water several times, and dried it by putting it into an oven and measured its mass. Then we made slurry by using 80% active material (Fe_3_O_4_, FeS, or FeS–Fe_3_O_4_ composite), 10% activated carbon (AC), and 10% polyvinylidene (PVDF) by adding *N*-methyl-2-pyrrolidone (NMP) drop-wise. Then, this slurry was coated onto the Ni-foam substrate and dried in an oven for 12 h at 60 °C. To further enhance the conductivity of the porous Ni-foam, the prepared electrode was pressed under a pressure of 100 kPa for 2 min. The mass of the active material was determined by measuring the difference in weight of the Ni-foam substrate before and after electrode fabrication (for details see Fig. 1).

### 2.6 Characterization

The successful synthesis of Fe_3_O_4_, FeS, and FeS–Fe_3_O_4_ composites was confirmed by X-ray diffraction (XRD) analysis using a Donghua TD-3500 diffract meter over a 2*θ* range of 20°–70°. The morphology of the samples was examined *via* Field Emission Scanning Electron Microscopy (FESEM) using a JEOL JSM-7800F instrument. The electrochemical performance, cyclic voltammetry (CV), galvanostatic charge discharge (GCD), and EIS, of the prepared electrodes was evaluated in a 3 M KOH aqueous electrolyte using a Donghua DH7000C workstation. A platinum wire and an Ag/AgCl electrode were employed as the counter and reference electrodes, respectively. After that an asymmetric supercapacitor were fabricated and its electrochemical performance was evaluated using FeS–Fe_3_O_4_ as the positive electrode and activated carbon (AC) as the negative electrode in 3 M KOH electrolyte and its stability was tested at 1.4 V over 12 000 charge–discharge cycles. Furthermore all other electrochemical parameters were calculated by using these equations.1
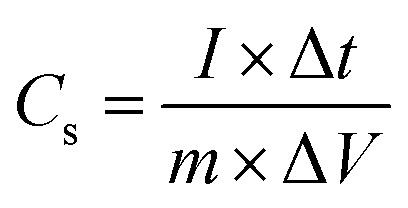
2
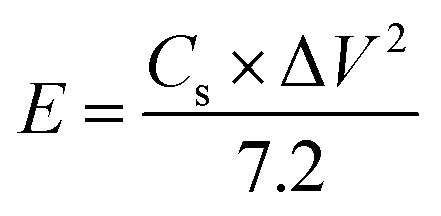
3
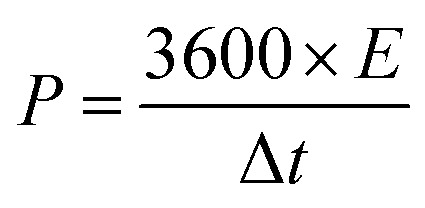
4
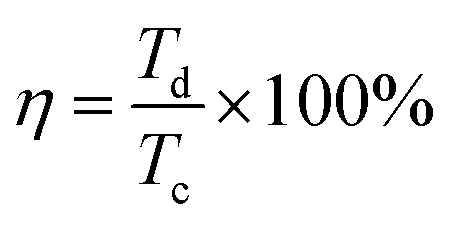
where *C*_s_ (F g^−1^) denotes the specific capacitance, *I*/*m* signifies the current density, Δ*V* represents the potential difference, Δ*t* indicates the discharge duration, and *E* (Wh kg^−1^) and *P* (W kg^−1^) correspond to energy and power density, respectively.

## 3. Results and discussion

### 3.1 Elemental analysis of prepared samples

**Fig. 2:**
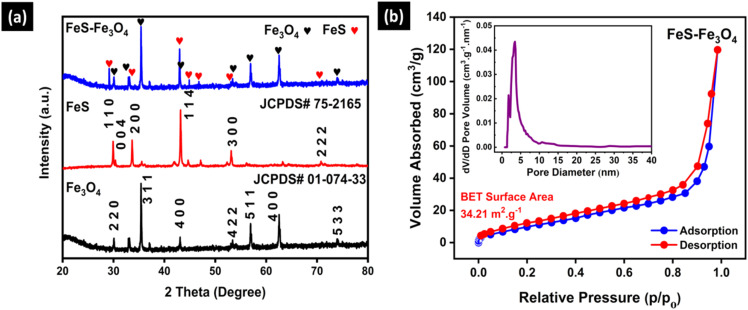
(a) X-ray diffraction of Fe_3_O_4_, FeS, and FeS–Fe_3_O_4_ composite. (b) BET analysis of the FeS–Fe_3_O_4_ composite.

The diffraction peaks of Fe_3_O_4_ matched with JCPDS # 01-074-33 showing peaks at positions 30°, 35°, 44°, 57°, and 64° corresponding to the respective *hkl* values (2 0 0), (3 1 1), (4 0 0), (5 1 1), and (4 4 0). The diffraction peaks of FeS matched with JCPDS # 75-2165, corresponding to the positions 29.9°, 34.7°, 43.2°, and 53.1°, having *hkl* values (1 1 0), (1 1 2), (1 0 5), and (3 0 0). The FeS–Fe_3_O_4_ composite shows all the distinct peaks of FeS, and Fe_3_O_4_, confirming the successful preparation of the composite material (Fig. 1a). Fig. 2b shows the BET surface area analysis and pore size distribution of the FeS–Fe_3_O_4_ nanocomposite. The adsorption–desorption isotherms show type IV behavior, which is typical of mesoporous materials. As the relative pressure approaches 1, the volume absorbed increases a lot. The BET surface area of the composite is predicted to be 34.21 m^2^ g^−1^, indicating a moderate surface area suitable for energy storage applications. The inset in Fig. 2b shows the pore size distribution, which is highest around 3–5 nm. This shows that the composite material has mesopores. These mesopores are useful for boosting the electrochemical performance of the FeS–Fe_3_O_4_ nanocomposite, as they provide additional surface area for charge storage. Nitrogen adsorption–desorption measurements reveal that both Fe_3_O_4_ and FeS exhibit typical type-IV isotherms with pronounced hysteresis loops, indicating their mesoporous nature. The BET surface areas of Fe_3_O_4_ (24.3 m^2^ g^−1^) and FeS (28.54 m^2^ g^−1^) confirm moderately porous structures that facilitate electrolyte penetration and ion transport; further details are provided in the SI (Fig. S3a and b).

**Fig. 3:**
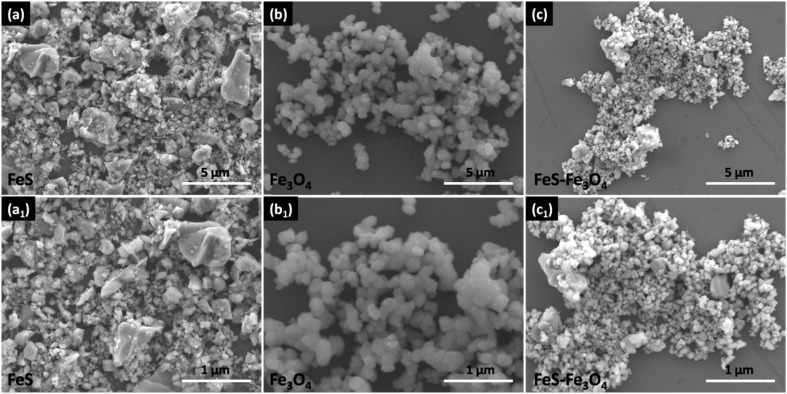
Field emission scanning electron microscopy of FeS (a and a_1_), Fe_3_O_4_ (b and b_1_), and FeS–Fe_3_O_4_ composite (c and c_1_).

Fig. 3(a and a_1_) shows the morphology of FeS, which exhibits distributed nanoparticles with rough surfaces; the particles show agglomeration with sharp edges, and the agglomerates show strong inter-particle interactions with limited porosity within the FeS structure. Fig. 3(b and b_1_) display the SEM images of Fe_3_O_4_, revealing a relatively uniform morphology composed of near-spherical nanoparticles. These particles are closely packed and interconnected, forming chain-like and clustered assemblies. Compared to FeS, Fe_3_O_4_ exhibits a more homogeneous particle size distribution and smoother surfaces, indicating controlled nucleation and growth during synthesis. The interconnected nanoparticle network is beneficial for facilitating electron transport and structural stability. Fig. 3(c and c_1_) shows the morphology of the FeS–Fe_3_O_4_ composite, where a distinct hybrid structure can be clearly observed. The composite displays a hierarchical and porous architecture formed by the intimate integration of FeS and Fe_3_O_4_ particles. The Fe_3_O_4_ nanoparticles are uniformly anchored onto the FeS matrix, resulting in a rough and highly textured surface with abundant interparticle voids. This interconnected and porous morphology provides an increased surface area and more accessible active sites, which can effectively enhance electrolyte penetration and ion diffusion. The strong interfacial contact between FeS and Fe_3_O_4_ is expected to improve charge transfer kinetics and structural integrity during electrochemical cycling. So, the FESEM analysis confirms the successful formation of the FeS–Fe_3_O_4_ composite with a well-integrated hierarchical morphology, which is advantageous for electrochemical energy storage applications due to its enhanced surface area, improved conductivity pathways, and robust structural framework.

**Fig. 4:**
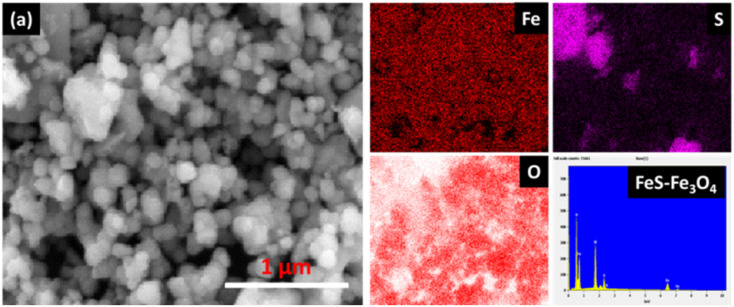
(a) EDS mapping of the FeS–Fe_3_O_4_ composite with EDX attachment.

Fig. 4a depicts the elemental representation of the FeS–Fe_3_O_4_ composite, with unique colors denoting all pertinent components, signifying the successful synthesis of the composite. The results are shown in dark red (Fe), purple (S), and blood red (O), showing the uniform distribution of the FeS–Fe_3_O_4_ composite. The last figure shows the EDX analysis of the FeS–Fe_3_O_4_ composite, confirming the successful formation of the FeS–Fe_3_O_4_ composite without any other additional peaks.

## 4. Electrochemical performance of FeS, Fe_3_O_4_, and FeS–Fe_3_O_4_ composite in a 3E system

**Fig. 5:**
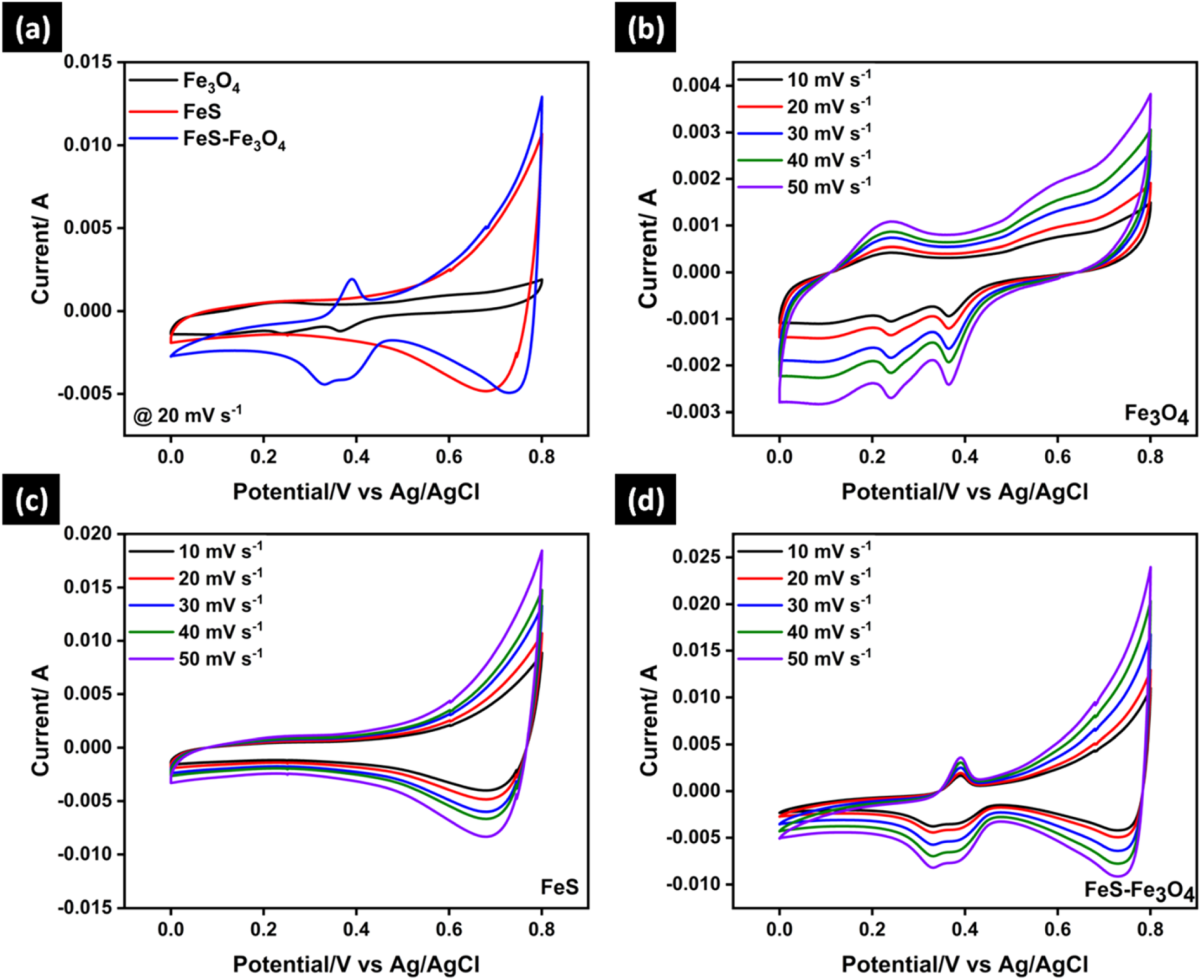
(a) Comparative CV analysis of Fe_3_O_4_, FeS, and FeS–Fe_3_O_4_ composite at 20 mV s^−1^, and CV analysis of (b) Fe_3_O_4_, (c) FeS, and (d) FeS–Fe_3_O_4_ nanocomposite at scan rates of 10 to 50 mV s^−1^.

Fig. 5a depicts a comparative cyclic voltammetry (CV) analysis of FeS, Fe_3_O_4_, and the FeS–Fe_3_O_4_ nanocomposite obtained at a scan rate of 20 mV s^−1^. Fig. 5(b–d) further demonstrate the CV curves of Fe_3_O_4_, FeS, and the FeS–Fe_3_O_4_ nanocomposite, respectively, obtained at various scan rates ranging from 10 to 50 mV s^−1^ within a potential window of 0.0–0.8 V. The FeS–Fe_3_O_4_ nanocomposite has a CV profile that is almost rectangular even at a high scan rate 50 mV s^−1^. This shows that it has excellent capacitive performance and fast charge transfer kinetics. In contrast, the CV curves of pure FeS and Fe_3_O_4_ electrodes exhibit strong redox peaks and obvious distortions, indicating sluggish reaction kinetics and poor electrochemical reversibility during the redox process. The composite electrode FeS–Fe_3_O_4_ shows a large area under the curve, which means its conductivity is better than that of both pure Fe_3_O_4_ and FeS electrodes. The reason for the large area under the curve is that FeS is more conductive than Fe_3_O_4_, which provides more electrons during the CV test Fig. 5a. The results demonstrate that both pure FeS and Fe_3_O_4_ electrodes have different redox peaks centered around 0.4 V, which are associated with the reversible Fe^2+^/Fe^3+^ redox processes.^47^ The fact that these acute redox patterns are less pronounced in the composite further supports its better capacitive properties and faster electrochemical responsiveness. The following reactions are involved in the 3 M KOH solution during the CV test.

5 FeS + 10OH^−^ → Fe(OH)_2_ + SO_4_^−2^ + 4H_2_O + 8e^−^

6 FeS + 8OH^−^ → Fe(OH)_2_ + SO_3_^−2^ + 3H_2_O + 6e^−^

7 Fe_3_O_4_ + H_2_O + (OH)^−^ → 3FeO(OH)|4K^+^ (Oxidation)

8 3FeO(OH) → Fe_3_O_4_ + H_2_O|4(OH)^−^ + 4K^+^ → 4KOH (Reduction)

In an alkaline electrolyte, reversible redox reactions involving both the FeS and Fe_3_O_4_ phases dictate the electrochemical characteristics of the FeS–Fe_3_O_4_ composite electrode. Eqn (7) and (8) show that the oxidation and reduction processes take place during the cyclic voltammetry analysis. In this reaction, FeS reacts with OH^−^ hydroxide ions to make iron hydroxide Fe (OH)_2_. At the same time, sulfur is transformed into SO_4_^2−^ and electrons are released. When FeS reacts in KOH, it forms Fe (OH)_2_ and oxidized sulfur, due to which at the electrode, the faradaic redox reaction occurs and the electrode acts like a pseudocapacitor, increasing the overall capacitance of the electrode.

The Fe_3_O_4_ phase also plays a big role in charge storage by using iron oxy-hydroxide species in reversible oxidation–reduction processes. Fe_3_O_4_ oxidizes in the presence of water and hydroxide ions, as shown in eqn (7), forming FeO (OH) and releasing electrons. This process involves the oxidation of Fe^2+^ to Fe^3+^, which is a very important part of the faradaic charge-storage mechanism. During the cathodic scan, the reverse reaction happens, as given in eqn (8), where FeO (OH) is reduced back to Fe_3_O_4_ with the consumption of electrons and hydroxide ions, regenerating KOH in the electrolyte. This reversible transition between Fe_3_O_4_ and FeO (OH) ensures good redox reversibility and cycling stability.

The capacitive and diffusion-controlled charge storage contributions of the Fe_3_O_4_, FeS, and FeS–Fe_3_O_4_ composite electrodes were quantitatively examined at varied scan rates ranging from 10–50 mV s^−1^, as reported in Table S1. For the Fe_3_O_4_ electrode, the charge storage behavior is largely dominated by diffusion-controlled mechanisms at all scan rates. At a low scan rate 10 mV s^−1^, the diffusion contribution reaches 87%, while the capacitive contribution accounts for just 13%. With increasing scan rate, the capacitive contribution gradually improves to 39% at 50 mV s^−1^, indicating enhanced surface-controlled kinetics; nonetheless, diffusion-controlled behavior remains dominant, revealing the bulk redox nature of Fe_3_O_4_. In the case of the FeS electrode, a moderate increase in capacitive contribution is found compared to Fe_3_O_4_. At a scan rate of 10 mV s^−1^, the capacitive contribution is 22%, increasing steadily to 48% at 50 mV s^−1^, whereas the diffusion contribution declines from 78% to 52%. This pattern shows that FeS exhibits mixed charge storage behavior, where both surface redox reactions and diffusion-limited processes contribute significantly, perhaps because to its increased electrical conductivity relative to Fe_3_O_4_. Notably, the FeS–Fe_3_O_4_ nanocomposite electrode displays a much larger capacitive contribution across all scan rates. At 10 mV s^−1^, the capacitive contribution already reaches 34%, which further increases to 66% at 50 mV s^−1^, while the diffusion contribution similarly reduces from 66% to 34%. The relationship was utilized to precisely quantify the contributions of diffusion-controlled and capacitive processes.

9 *i*(*V*) = *K*_1_*v* + *K*_2_*v*^1/2^

This equation indicates that the surface capacitive effects are represented by *K*_1_*v*, whereas the diffusion-controlled insertion process is denoted by *K*_2_*v*_1/2_. Rearranging the sequence of the equation:10
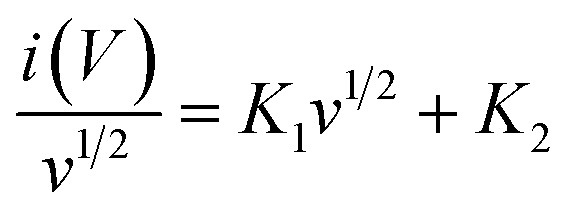


**Fig. 6:**
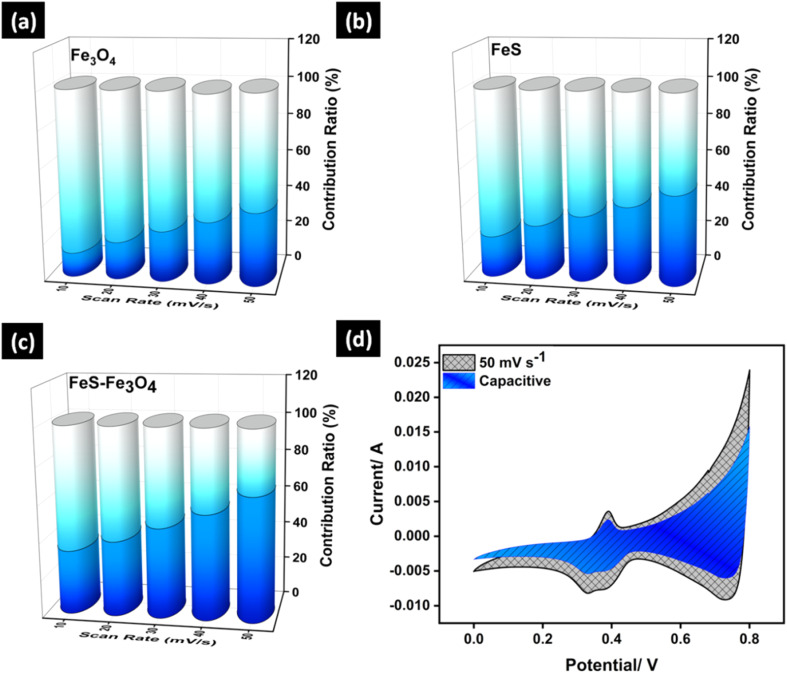
Capacitive–diffusion analysis of (a) Fe_3_O_4_, (b) FeS, and (c) FeS–Fe_3_O_4_ composite at 10–50 mV s^—1^ and (d) CV analysis of FeS–Fe_3_O_4_ at 50 mV s^−1^ with marked capacitive and diffusion contributions.

This dramatic shift toward capacitive-controlled behavior underlines the synergistic interaction between FeS and Fe_3_O_4_. The greater capacitive response can be due to enhanced electrical conductivity, increased electrochemically active surface area, and quicker ion transport at the hetero interface between the two phases. Overall, the FeS–Fe_3_O_4_ composite demonstrates a dominant surface-controlled charge storage mechanism at higher scan rates, which is particularly desirable for high-rate energy storage applications. The enhanced capacitive contribution verifies faster reaction kinetics and improved reversibility, explaining the superior electrochemical performance of the composite compared to the individual FeS and Fe_3_O_4_ electrodes (Fig. 6).

**Fig. 7:**
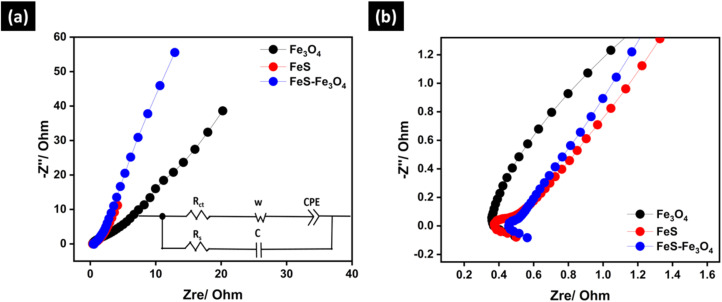
EIS analysis of (a) Fe_3_O_4_, FeS, and FeS–Fe_3_O_4_ composite, and the (b) zoomed-in view.

Fig. 7 shows the electrochemical impedance parameters found for the Fe_3_O_4_, FeS, and FeS–Fe_3_O_4_ composite electrodes. The three electrodes have nearly equal solution resistance (*R*_s_) values. This means that the electrolyte resistance is about the same and that the electrode materials and the current collector have good electrical contact. The *R*_s_ value for the Fe_3_O_4_ electrode is 0.49 Ω, while the *R*_s_ values for FeS and the FeS–Fe_3_O_4_ composite are 0.50 Ω and 0.56 Ω, respectively. There is a considerable difference in the charge transfer resistance (*R*_ct_) between the electrodes. The *R*_ct_ value of the Fe_3_O_4_ electrode is 2.34 Ω, which means that charge transfer at the point where the electrode and electrolyte come in contact is slow. The FeS electrode has a much lower *R*_ct_ of 0.54 Ω, indicating higher conductivity and faster electron transport between surfaces. The *R*_ct_ value of the FeS–Fe_3_O_4_ composite electrode is 0.55 Ω, which is much lower than that of pure Fe_3_O_4_ and similar to that of FeS. FeS serves as an efficient electronic pathway, ensuring rapid electron transport across the electrode, while also providing structural support that mitigates mechanical stress and suppresses agglomeration of Fe_3_O_4_ during cycling. Most importantly, the FeS–Fe_3_O_4_ composite induces interfacial polarization and electronic redistribution, creating abundant electrochemically active sites and accelerating interfacial redox reactions.^52^ The impedance results reveal that the creation of the FeS–Fe_3_O_4_ composite significantly boosts interfacial charge transfer while preserving low solution resistance, which leads to the higher electrochemical performance, observed for the composite electrode (Table 1).

**Table 1:** The *R*_s_, and *R*_ct_ values of Fe_3_O_4_, FeS, and FeS–Fe_3_O_4_ nanocomposite electrodes

Electrodes	*R*_s_ (Ω)	*R*_ct_ (Ω)
Fe_3_O_4_	0.49	2.34
FeS	0.50	0.54
FeS–Fe_3_O_4_ composite	0.56	0.55

**Fig. 8:**
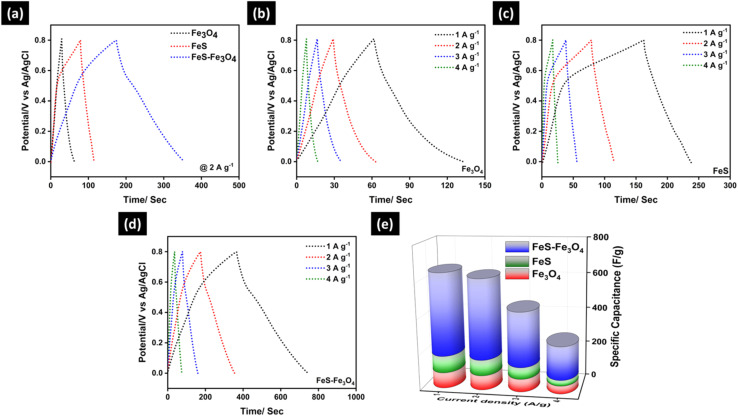
(a) Comparative GCD analysis of Fe_3_O_4_, FeS, and FeS–Fe_3_O_4_ composite at 2 A g^−1^, and GCD of (b) Fe_3_O_4_, (c) FeS, and (d) FeS–Fe_3_O_4_ composite at 1–5 A g^−1^, and (e) specific capacitance of all prepared electrodes at 1–5 A g^−1^.

Fig. 8 shows the charge–discharge behavior of Fe_3_O_4_, FeS, and the FeS–Fe_3_O_4_ composite electrodes in a 3 M KOH electrolyte. At a current density of 2 A g^−1^ and in a potential window of (0.0–0.8) V, Fig. 8a compares the GCD curves of the three electrodes. The FeS–Fe_3_O_4_ composite has a substantially longer discharge time than the other electrodes, which means that it can store more charge than pure Fe_3_O_4_ and FeS. This enhanced performance is due to the synergistic interaction between FeS and Fe_3_O_4_, which facilitates charge transfer and ion diffusion. Fig. 8(b–d) show the GCD curves for Fe_3_O_4_, FeS, and the FeS–Fe_3_O_4_ composite at current densities of 1, 2, 3, and 4 A g^−1^. The charge and discharge curves of the Fe_3_O_4_ electrode (Fig. 8b) are comparable to each other. But when the current density gets higher, the discharge time gets shorter, which indicates that the rate capability is limited. The FeS electrode in Fig. 8c shows a similar pattern. When the current density is higher, the discharge time is shorter, leading to higher polarization. This illustrates that electrochemical kinetics are limited. The GCD curves for the FeS–Fe_3_O_4_ composite electrode (Fig. 8d) are clear and highly symmetrical, even at high current densities. This means that it can be readily reversed and operates better at higher rates. The lower voltage drop and longer discharge time indicate that the composite structure has better electrical conductivity and faster ion transport. Fig. 8e summarizes the specific capacitance values of all electrodes calculated from the GCD curves at different current densities; for details, see Table 2. At 1 A g^−1^, the FeS–Fe_3_O_4_ composite delivers a high specific capacitance of 464.6 F g^−1^, which is substantially higher than that of Fe_3_O_4_ (88.13 F g^−1^) and FeS (94.5 F g^−1^). Even at higher current densities of 2, 3, and 4 A g^−1^, the composite retains capacitance values of 449.5, 307.1, and 186.9 F g^−1^, respectively, demonstrating superior rate capability. In comparison, both Fe_3_O_4_ and FeS electrodes suffer from a more pronounced decline in capacitance with increasing current density. The outstanding electrochemical performance of the FeS–Fe_3_O_4_ composite can be attributed to the combined effects of enhanced conductivity from FeS and the high pseudocapacitive contribution of Fe_3_O_4_, along with improved electrolyte accessibility and structural stability.

**Table 2:** Specific capacitance of Fe_3_O_4_, FeS, and FeS–Fe_3_O_4_ nanocomposite electrodes in 3 M KOH solutions

Current density A g^−1^	Fe_3_O_4_ F g^−1^	FeS F g^−1^	FeS–Fe_3_O_4_ F g^−1^
1	88.13	94.5	464.6
2	81.43	88.3	449.5
3	72.10	65.5	307.1
4	44.65	32.1	186.9

## 5. Electrochemical performance of the asymmetric FeS–Fe_3_O_4_//AC supercapacitor

**Fig. 9:**
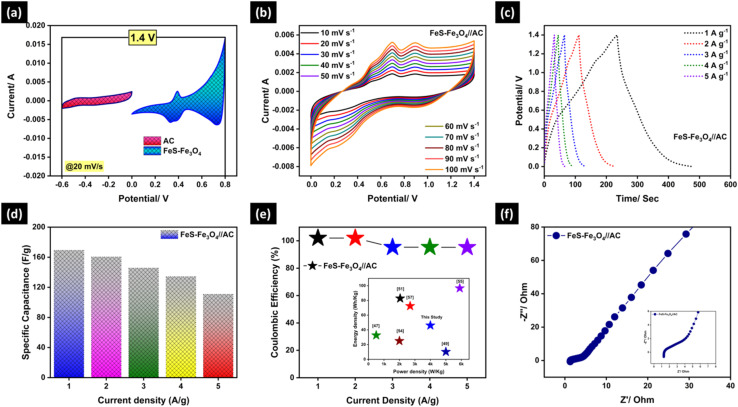
Asymmetric device analysis of FeS–Fe_3_O_4_//AC: (a) CV window of the FeS–Fe_3_O_4_ composite device, (b) CV analysis at 10–100 mV s^−1^, (c) GCD analysis at 1–5 A g^−1^, (d) specific capacitance of the tdevice, (e) coulombic efficiency ofhe device at 1–5 A g^−1^, and (f) EIS analysis.

Fig. 9 presents the electrochemical performance of the assembled asymmetric supercapacitor device based on the FeS–Fe_3_O_4_ composite as the positive electrode and activated carbon (AC) as the negative electrode, evaluated in a 3 M KOH electrolyte. Fig. 9a shows the CV window of both AC (−0.6 to 0.0) V and FeS–Fe_3_O_4_ composite (0.0 to 0.8) V at a scan rate of 20 mV s^−1^ in 3 M KOH solution. The CV shape of AC is rectangular, showing its EDLC behaviour, while the FeS–Fe_3_O_4_ composite shows pseudocapacitive behaviour with a broader response due to reversible redox reactions. The suitable window for both AC and FeS–Fe_3_O_4_ composite confirms the construction of the asymmetric device for practical applications. Fig. 9b shows the CV analysis of the FeS–Fe_3_O_4_//AC device at various scan rates ranging from 10–100 mV s^−1^ in a potential window of 0.0 to 1.4 V. As the scan rate increases from 10–100 mV s^−1^, the area under the curve also increases, which shows that the electrochemical performance can be reversed easily and the transportation of charge happens quickly. The shape of the CV remains the same at high scan rates, which shows the good stability of the FeS–Fe_3_O_4_//AC device in 3 M KOH solution. Fig. 9c shows the galvanostatic charge–discharge curve at a current density of 1–5 A g^−1^. The CD curve remains the same even at a very small voltage drop. This shows that the device has high coulombic efficiency and excellent reversibility over repeated cycles. Fig. 9d shows the particular capacitance values that were found from the GCD curves at various current densities. At 1 A g^−1^, the device has a high specific capacitance of 169.29 F g^−1^, which slowly drops to 110.85 F g^−1^ at 5 A g^−1^ (for details see Table 3). This shows that it can operate effectively at high current densities. As the current density goes up, the energy density goes down from 46.10 to 30.17 Wh kg^−1^, while the power density goes up from 699.6 to 3998.4 W kg^−1^. This shows that the energy–power performance is very well balanced. Fig. 9e shows the coulombic efficiency of the FeS–Fe_3_O_4_//AC device at different current densities. The device has a high coulombic efficiency of about 102% at low current densities and it remains above 95% even at higher current densities. This means that it can charge and discharge quickly and doesn't lose much energy while it works. Fig. 9f shows the EIS analysis performed for the FeS–Fe_3_O_4_//AC device. The Nyquist plot shows that *R*_s_ is 1.27 ohms, which shows that the 3 M KOH electrolyte has strong ionic conductivity with low internal resistance. The low charge transfer resistance (*R*_ct_) of 3.96 ohm shows fast electron transport inside the device. So, from all the above results, it is clear that the FeS–Fe_3_O_4_//AC device is a promising device for energy storage applications.

**Table 3:** Specific capacitance, energy density, power density, and coulombic efficiency of the asymmetric FeS–Fe_3_O_4_//AC device at a current density of 1–5 A g^−1^

Current density (A g^−1^)	Specific capacitance (F g^−1^)	Energy density (Wh kg^−1^)	Power density (W kg^−1^)	Coulombic efficiency (%)
1	169.29	46.10	699.6	102
2	160.50	43.62	1401.1	102
3	145.71	39.59	2098.4	95.3
4	134.28	36.45	2801.2	95.3
5	110.85	30.17	3998.4	95.2

**Fig. 10:**
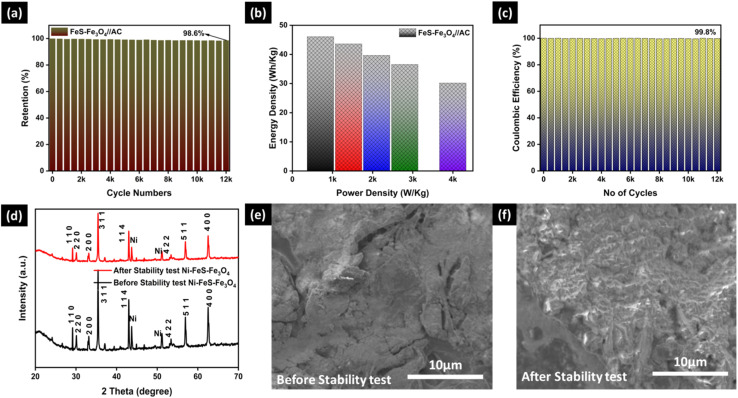
(a) Cycling stability of the Fes–Fe_3_O_4_//AC asymmetric device over 12k cycles at 7 A g^−1^, (b) energy density *vs.* power density, (c) coulombic efficiency over 12k cycles, (d) XRD analysis of FeS–Fe_3_O_4_ before and after the stability test, (e) SEM before the stability test, and (f) SEM after the stability test.

Fig. 10 shows the cycling stability, energy density *vs.* power density, and coulombic efficiency of the prepared asymmetric device (FeS–Fe_3_O_4_//AC). Fig. 10a shows the cycling stability of the asymmetric device over 12 000 continuous charge–discharge cycles at a current density of 7 A g^−1^. The device retains 98.6% of its initial capacitance after prolonged cycling, demonstrating its excellent electrochemical durability and structural stability. The high retention confirms that the FeS–Fe_3_O_4_ nanocomposite and AC maintain strong interfacial integrity during the repeated redox reactions. Fig. 10b shows the energy density and power density of the asymmetric device at a current density of 1–5 A g^−1^. The device attains a highest energy density of 46.10 Wh kg^−1^, with a power density of 699.6 W kg^−1^. At high current density, the energy density reaches a value of 30.17 Wh kg^−1^, and power density reaches 3998.4 W kg^−1^, highlighting its excellent rate capability. This favorable balance between energy and power densities reflects the synergistic contribution of the pseudocapacitive FeS–Fe_3_O_4_ composite and the electric double-layer behavior of the AC electrode, making the device suitable for high-power energy storage applications. Fig. 10c shows how well the FeS–Fe_3_O_4_//AC device works after 12 000 charge–discharge cycles. During the entire cycling test, the coulombic efficiency remains rather steady at about 99.8%. This indicates that electrochemical reactions are highly reversible and involve very little energy (more details can be seen in Table S2). The asymmetric device's extraordinary efficiency further confirms its superb charge transfer kinetics and long-term stability. These findings demonstrate the FeS–Fe_3_O_4_//AC asymmetric supercapacitors' energy–power characteristics, high efficiency, and great cycle stability. This shows that it has a lot of potential for use in the future as a way to store energy in a way that doesn't harm the environment. Fig. 10d displays the XRD analysis of the FeS–Fe_3_O_4_ composite before and after the stability test. The peaks of the FeS–Fe_3_O_4_ composite matched with the characteristic planes of FeS and Fe_3_O_4_, which confirms the successful formation of the composite. Two peaks are also observed, which belong to Ni-foam. After the stability test, the intensity reduced slightly, but all the peaks confirm the good crystallinity of the FeS–Fe_3_O_4_ composite. Fig. 10(e and f) shows the SEM image of the FeS–Fe_3_O_4_ composite on Ni-foam before and after the stability test at 10 µm. The electrode before the stability test shows a comparatively smooth surface, and after the stability test, it shows a well distributed active material that provides sufficient active sites for efficient ion transfer in 3 M KOH electrolyte. The FESEM image after the stability test shows that the overall morphology of the FeS–Fe_3_O_4_ composite is well preserved. Although minor surface roughening and agglomeration of particles are observed, no structural collapse occurs. The maintained morphology after 12 K cycles further confirms the mechanical robustness and electrochemical performance of the FeS–Fe_3_O_4_ composite electrode.

## 6. Conclusion

In summary, a FeS–Fe_3_O_4_ nanocomposite was successfully synthesized and systematically investigated as an efficient electrode material for advanced supercapacitor applications. Structural and morphological characterization confirmed the formation of a well-integrated heterostructure composed of crystalline FeS and Fe_3_O_4_ phases, providing abundant electroactive sites and a favourable architecture for rapid ion transport. Electrochemical evaluations demonstrated that the FeS–Fe_3_O_4_ composite delivers significantly enhanced capacitive performance compared to the individual FeS and Fe_3_O_4_ electrodes, achieving a high specific capacitance of 464.6 F g^−1^ at 1 A g^−1^ and maintaining good rate capability at higher current densities. The improved electrochemical behaviour is primarily attributed to the synergistic interaction between the high electrical conductivity of FeS and the strong pseudocapacitive contribution of Fe_3_O_4_, which together facilitate fast charge transfer and efficient redox reactions. Furthermore, an asymmetric supercapacitor device assembled using the FeS–Fe_3_O_4_ nanocomposite as the positive electrode and activated carbon as the negative electrode operated stably over an extended voltage window of 1.4 V in 3 M KOH electrolyte. The device exhibited a high energy density of 46.10 Wh kg^−1^ at a power density of 699.6 W kg^−1^ and retained 30.17 Wh kg^−1^ at a high power density of 3998.4 W kg^−1^. Remarkably, the device maintained 98.6% capacitance retention and a coulombic efficiency of approximately 99.8% after 12 000 charge–discharge cycles, highlighting its excellent long-term stability and reversibility. Overall, this study demonstrates that the FeS–Fe_3_O_4_ nanocomposite is a promising electrode material for high-performance, durable, and practical energy storage systems.

## Author contributions

Junaid Riaz: wrote the original manuscript draft, data creation, characterization, and electrochemical analysis. Muhammad Arif: supervision, review and editing, formal analysis. Ghulam Murtaza: software analysis. Zahra Bayhan: review and editing, Amina Bibi: supervision, review and editing, characterization, and formal analysis.

## Conflicts of interest

The authors declare that they have no conflict of interest.

## Supplementary Material

NA-008-D5NA01165E-s001

## Data Availability

The data that have been used are confidential & will be available on request. Supplementary information (SI) is available. See DOI: https://doi.org/10.1039/d5na01165e.
