# Supplementary material for: Facile synthesis of FeS–Fe3O4 nanocomposites: highly stable & enhanced electrochemical performance in asymmetric supercapacitor applications
Source: Nanoscale Adv. 2026 Feb 17;8(7):2364–77. doi: 10.1039/d5na01165e (PMC12964265; doi:10.1039/d5na01165e)
Supplement: NA-008-D5NA01165E-s001 [file NA-008-D5NA01165E-s001.pdf]

Supplementary Information (SI) for Nanoscale Advances.
This journal is © The Royal Society of Chemistry 2026

Supporting Information

Facile synthesis of FeS-Fe_3O_4 nanocomposite: Highly stable & enhanced electrochemical performance in Asymmetric Supercapacitor Applications

Junaid Riaz [1], Zahra Bayhan [2], Ghulam Murtaza [3], Muhammad Arif [1*], Amina Bibi [4**]

Authorship: Junaid Riaz

Co-Authorship: Zahra Bayhan and Ghulam Murtaza

Corresponding Authorship: Muhammad Arif , and Amina Bibi

[1] *Yunnan Key Laboratory of Optoelectronic Information Technology, School of Physics and Electronic Information, Yunnan Normal University, Kunming 650500, China.*

[2] *Department of Physics, College of Sciences, Princess Nourah bint Abdulrahman University, P.O. Box 84428, Riyadh 11671, Saudi Arabia.*

[3] *School of Ecology and Environmental Science, Yunnan University, Biocontrol Engineering Research center of Crop Diseases & Pests, Yunnan Province, Kunming 650500, China.*

[4] *Department of Physics, Hazara University, Mansehra 21300, Pakistan.*

Email: junaidriaz1990@gmail.com, aminaamni11@gmail.com,

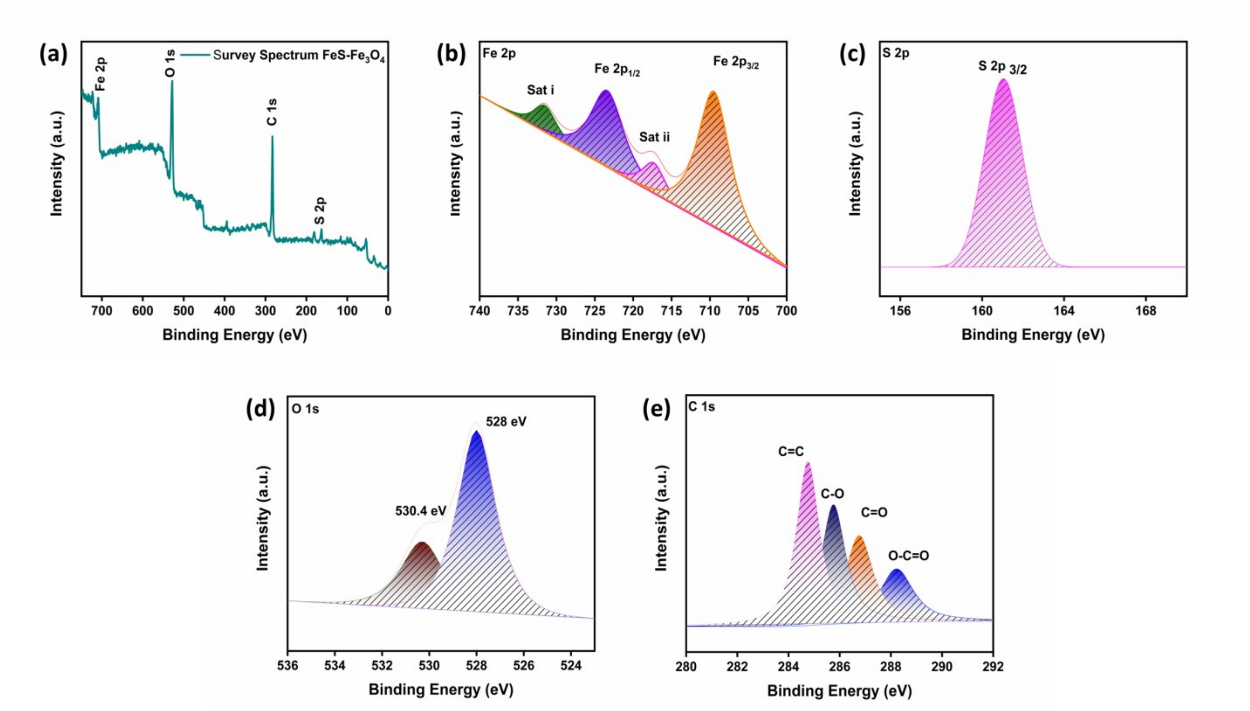

Fig S1. XPS analysis of FeS-Fe_3O_4 composite, (a) Survey Spectrum of FeS-Fe_3O_4, (b) Fe 2p, (c) S 2p, (d) O 1s, and (e) C 1s

The figure presents XPS data of FeS-Fe_3O_4 composite materials, highlighting the changes in the valence states of the elements and their impact on the electrochemical performance. In the survey spectrum (a), the prominent peaks indicate the presence of iron (Fe), sulfur (S), oxygen (O), and carbon (C), confirming the successful synthesis of the composite. The Fe $_{2p}$ spectrum (b) reveals a mix of Fe^{2+} and Fe^{3+} oxidation states, which are characteristic of the FeS and Fe_3O_4 phases, respectively. This mixed valence state is significant because it enhances the electrochemical activity, as both iron species contribute to charge storage and cycling stability. In the S 2p spectrum (c), S is primarily present in the S^{2-} state, which is expected for the FeS phase, playing a crucial role in improving the composite's charge storage capability. The O 1s spectrum (d) shows two peaks, one at 528 eV corresponding to oxygen in the Fe_3O_4 phase and another at 530.4 eV associated with surface-adsorbed oxygen or oxygen in the oxide phase. These oxygen states are vital for enhancing the conductivity and overall electrochemical behavior of the material. The C 1s spectrum (e) shows a peak at 283.88 eV, typical of surface contamination or the presence of a binder, confirming the presence of carbon in the material. The interface between the FeS and Fe_3O_4 phases plays a crucial role in the electrochemical performance of the

composite. The mixed oxidation states at the interface enhance electron and ion conductivity, which is essential for improving the cycling stability and rate performance of supercapacitors. The combination of these two materials leads to a synergistic effect, where the properties of each phase complement each other, resulting in improved charge transfer, better cycling stability, and enhanced energy storage capabilities compared to individual materials. This makes the FeS-Fe_3O_4 composite a promising candidate for supercapacitor applications.

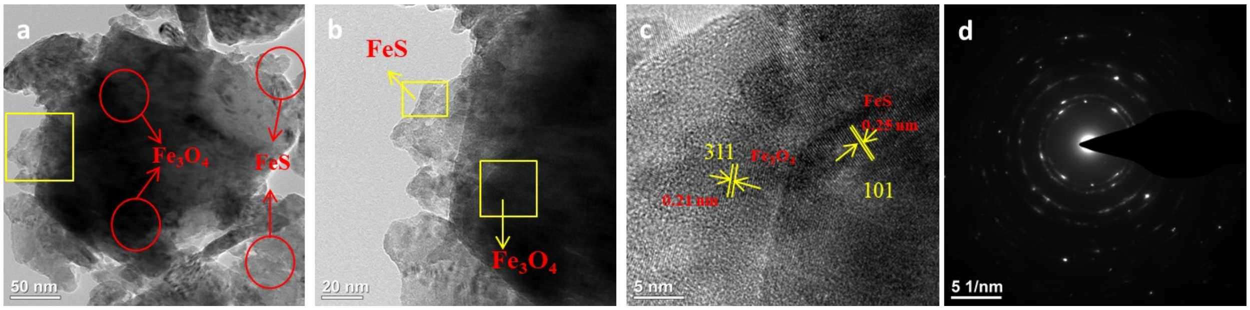

Fig S2. TEM analysis of FeS-Fe_3O_4 composite with SEAD

Figure S2 presents a comprehensive structural and compositional study of the composite material via transmission electron microscopy (TEM). Fig S2 (a) provides a low-magnification overview (50 nm scale bar), illustrating the general distribution of the two constituent phases. The Fe_3O_4 and FeS domains are clearly delineated with red circles, affirming their coexistence and illustrating their spatial configuration inside the composite. At a finer scale, Fig S2 (b) displays a high-magnification image (20 nm scale bar) in which the interface between Fe_3O_4 and FeS is distinctly delineated. This perspective enables us to analyze the close contact and boundary areas between the two phases, which is crucial for comprehending their synergistic interaction and interfacial charge transfer mechanisms. The crystalline detail is discerned in the high-resolution TEM picture presented in Fig S2 (c) (5 nm scale bar). Distinct lattice fringes are observable, corresponding to the (3 1 1) planes of Fe_3O_4 with an interplanar distance of 0.29 nm, and the (1 0 1) planes of FeS with a distance of 0.25 nm. This image validates the high crystallinity of both components and displays the alignment of their crystal lattices at the heterojunction, which is essential for promoting efficient electron transport and stability during electrochemical cycling. Ultimately, Fig S2 (d) presents the Selected Area Electron Diffraction (SAED) pattern, with a scale bar of 5.1 nm. The concentric diffraction rings correspond to specific crystallographic planes of Fe_3O_4 and FeS, offering conclusive confirmation of the composite's polycrystalline

origin and further confirming the phase purity and structural order noted in the direct imaging modes.

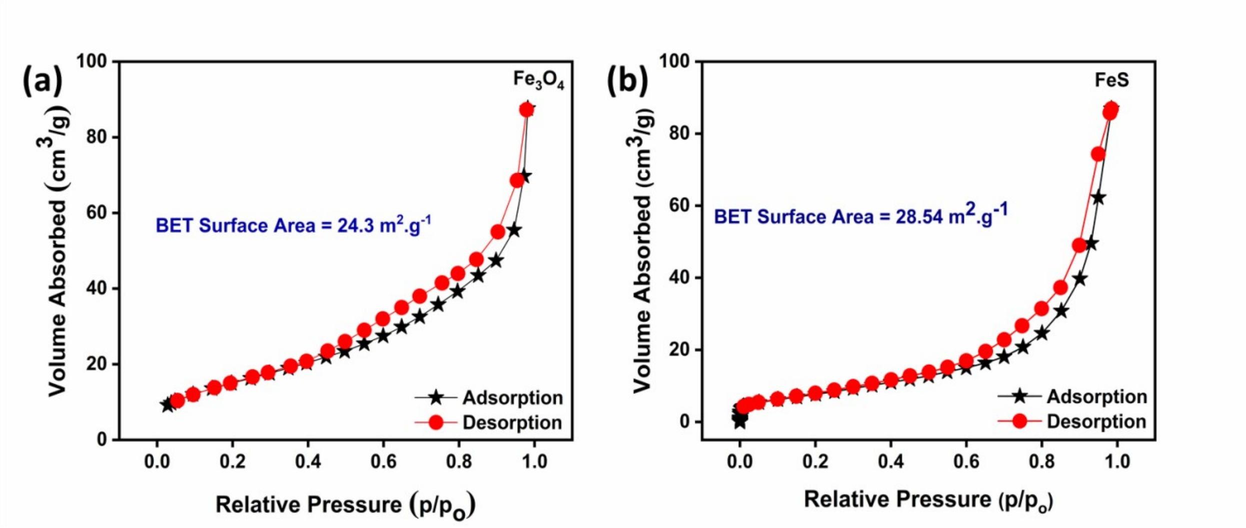

Fig S3. BET analysis of (a) Fe_3O_4, and (b) FeS

Figures S3 (a) and (b) show the nitrogen adsorption-desorption isotherms of Fe_3O_4 and FeS, respectively. These are utilized to test their surface textural qualities using BET analysis. In Fig. (a), the Fe_3O_4 sample shows a classic type-IV isotherm with a visible hysteresis loop between the adsorption and desorption branches at intermediate to high relative pressures (p/p_0). This means that there are mesoporous structures present. The steady rise in adsorbed volume at low p/p_0 indicates monolayer–multilayer adsorption, but the rapid absorption at elevated relative pressures is ascribed to capillary condensation within mesopores. The BET surface area of Fe_3O_4 is 24.3 m^2 g^{-1}, which means that it has a moderately porous structure that can provide electroactive areas that are easy to reach. Similarly, Fig. S3 (b) shows the N_2 adsorption–desorption isotherm of FeS, which also follows a type-IV profile with a pronounced hysteresis loop, confirming its mesoporous nature. Compared to Fe_3O_4, FeS demonstrates a slightly higher nitrogen uptake over the entire pressure range, resulting in a larger BET surface area of 28.54 m^2 g^{-1}. This increased surface area implies a higher density of exposed active sites and more efficient electrolyte penetration. Overall, the mesoporous characteristics and relatively high specific surface areas of both Fe_3O_4 and FeS are favorable for enhancing ion diffusion and charge storage kinetics, which are crucial for improved electrochemical performance when these materials are employed individually or as part of a composite electrode system.

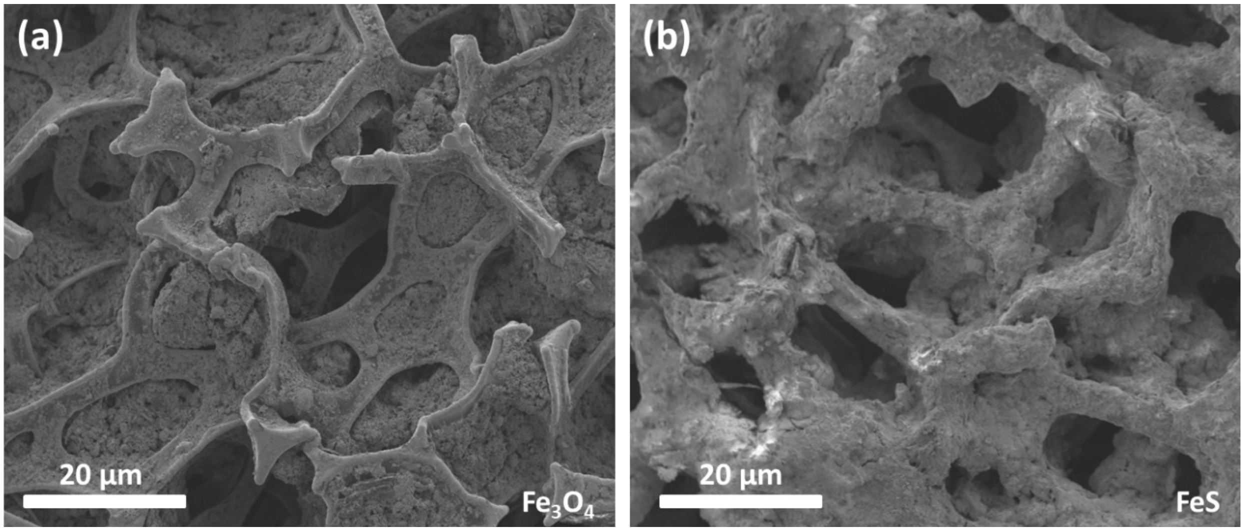

Fig S4. SEM analysis of (a) Fe_3O_4 after electrochemical performance, and (b) FeS after electrochemical performance

Table S1: Capacitive and Diffusion contribution of Fe_3O_4, FeS, and FeS-Fe_3O_4 composite

**Scan Rate ($mV \cdot s^{-1}$)**	**Fe_3O_4 Electrode**		**FeS Electrode**		**FeS- Fe_3O_4 composite Electrode**	
	Capacitive %	Diffusion %	Capacitive %	Diffusion %	Capacitive %	Diffusion %
10	13	87	22	78	34	66
20	20	80	29	71	40	60
30	27	73	35	65	48	52
40	33	66	41	59	56	44
50	39	61	48	52	66	34

Table S2: Comparative analysis of FeS and Fe_3O_4 with different composite

**Electrode Materials**	**Current density ($A \cdot g^{-1}$)**	**Specific Capacitance ($F \cdot g^{-1}$)**	**Energy density ($Wh \cdot kg^{-1}$)**	**Power density ($W \cdot kg^{-1}$)**	**No of cycles @ retention**	**Ref.**
Fe_3O_4@C@PANI	0.5	420	32.7	500	5000@ 78%	[1]
Fe_2O_3-Fe_3O_4@C	5	274	N/A	N/A	5000@ 83%	[2]
Au-Fe_3O_4	1	464	10.22	5000	1000@ 72.8	[3]
Fe_3O_4/rGO	1 $mV \cdot s^{-1}$	350	N/A	N/A	10,000@ 100%	[4]
G@Fe_3O_4	2	732	82.8	2047	10,000@ 88.3%	[5]
Fe_3O_4 NP	0.5	195.18	N/A	N/A	3000@ 94%	[6]

Fe_3O_4/rGO	5	315	N/A	N/A	2000@ 95%	[7]
Fe_3O_4 NP	10 $mV{\cdot}s^{-1}$	91.82	24.99	2000	1000@≥60%	[8]
Fe_3O_4@SiO_2@Fc	2.5	161$mAh{\cdot}g^{-1}$	96.6	5896	3000@84.6%	[9]
NiO/Fe_3O_4/rGO	N/A	1155	N/A	N/A	10,000@90.6 %	[10]
NiO-C@Fe_3O_4	1	1210	72.49	2690.35	10,000@83.3 2%	[11]
FeS-Fe_3O_4	1	464.6	46.10	3998.4	12000@ 98.6%	**This Study**

References:

[1] Qiu, Z., Peng, Y., He, D., Wang, Y., & Chen, S. (2018). Ternary Fe3O4@ C@ PANi nanocomposites as high-performance supercapacitor electrode materials. *Journal of materials science*, *53*(17), 12322-12333.

[2] Arun, T., Prabakaran, K., Udayabhaskar, R., Mangalaraja, R. V., & Akbari-Fakhrabadi, A. (2019). Carbon decorated octahedral shaped Fe3O4 and α-Fe2O3 magnetic hybrid nanomaterials for next generation supercapacitor applications. *Applied Surface Science*, *485*, 147-157.

[3] Liu, S., Guo, S., Sun, S., & You, X. Z. (2015). Dumbbell-like Au-Fe 3 O 4 nanoparticles: a new nanostructure for supercapacitors. *Nanoscale*, *7*(11), 4890-4893.

[4] Qi, T., Jiang, J., Chen, H., Wan, H., Miao, L., & Zhang, L. (2013). Synergistic effect of Fe3O4/reduced graphene oxide nanocomposites for supercapacitors with good cycling life. *Electrochimica Acta*, *114*, 674-680.

[5] Lin, J., Liang, H., Jia, H., Chen, S., Guo, J., Qi, J., ... & Feng, J. (2017). In situ encapsulated Fe_3O_4 nanosheet arrays with graphene layers as an anode for high-performance asymmetric supercapacitors.

[6] Aghazadeh, M., Karimzadeh, I., & Ganjali, M. R. (2017). Electrochemical evaluation of the performance of cathodically grown ultra-fine magnetite nanoparticles as electrode material for supercapacitor applications. *Journal of Materials Science: Materials in Electronics*, *28*(18), 13532-13539.

[7] Das, A. K., Sahoo, S., Arunachalam, P., Zhang, S., & Shim, J. J. (2016). Facile synthesis of Fe 3 O 4 nanorod decorated reduced graphene oxide (RGO) for supercapacitor application. *RSC advances*, *6*(108), 107057-107064.

[8] Saini, P., Yadav, J. K., Rani, B., Pandey, A. P., & Dixit, A. (2024). High-performance symmetric supercapacitor using cost-efficient iron oxide (Fe3O4) nanoparticles. *Energy Storage*, *6*(2), e608.

[9] Payami, E., Mohammadzadeh, A., Safa, K. D., & Teimuri-Mofrad, R. (2024). Ferrocene surface-modified Fe3O4 nanoparticles as prominent electrode material for supercapacitor application. *Journal of Energy Storage*, *88*, 111624.

[10] Askari, M. B., Moghadam, M. T. T., & Salarizadeh, P. (2024). Three-component NiO/Fe3O4/rGO nanostructure as an electrode material towards supercapacitor and alcohol electrooxidation. *Heliyon*, *10*(20).

[11] Hanamantrao, D. P., Kasiviswanathan, K., Kumaresan, L., Littleflower, S. R. S., Ramakrishnan, S., Rangasamy, B., & Vediappan, K. (2024). Ultrahigh faradaic NiO anchored carbon-coated Fe-MOF derived Fe3O4 NiO-C@ Fe3O4 as potential electrode for pouch-type asymmetric supercapacitor. *Journal of Energy Storage*, *91*, 112118.
